# Residual Stress Analysis at the Conductor–Insulator Interface During the Curing Process of Hair-Pin Motors

**DOI:** 10.3390/polym16243514

**Published:** 2024-12-17

**Authors:** Mingze Ma, Hongyi Gan, Xiao Shang, Linsen Song, Yiwen Zhang, Jingru Liu, Chunbai Liu, Yanzhong Hao, Xinming Zhang

**Affiliations:** 1 School of Mechanical and Electrical Engineering, Changchun University of Science and Technology, Changchun 130022, China; mmz195797@163.com (M.M.); zxm@fosu.edu.cn (X.Z.); 2 FAW Tooling Die Manufacturing Co., Ltd., Changchun 130000, China; ganhongyi@faw.com.cn (H.G.); shangxiao_td@faw.com.cn (X.S.); liuqr_td@faw.com.cn (J.L.); 3 University-Enterprise Joint Innovation Laboratory of Intelligent Manufacturing, Assembly and Inspection Technology and Equipment for Automotive Parts and Components, Changchun University of Science and Technology, Changchun 130022, China; 4 FAW Casting Co., Ltd., Changchun 130013, China; liuchunbai@faw.com.cn (C.L.); haoyanzhong@faw.com.cn (Y.H.); 5 School of Mechatronic Engineering and Automation, Foshan University, Foshan 528225, China

**Keywords:** curing residual stress, unsaturated polyester imide resin (UPIR), curing kinetics, finite element analysis, hairpin motors

## Abstract

The curing process of hair-pin motor stator insulation is critical, as residual stress increases the risk of partial discharge and shortens a motor’s lifespan. However, studies on the stress-induced defects during insulation varnish curing remain limited. This research integrates three-dimensional numerical simulations and experimental analysis to develop a curing model based on unsaturated polyester imide resin, aiming to explore the mechanisms of residual stress formation and optimization strategies. A dual fiber Bragg grating (FBG) sensor system is employed for simultaneous temperature and strain monitoring, while curing kinetics tests confirm the self-catalytic nature of the process and yield the corresponding kinetic equations. The multi-physics simulation model demonstrates strong agreement with the experimental data. The results show that optimizing the curing process reduces the maximum stress from 45.1 MPa to 38.6 MPa, effectively alleviating the stress concentration. These findings highlight the significant influence of the post-curing temperature phase on residual stress. The proposed model offers a reliable tool for stress prediction and process optimization in various insulating materials, providing valuable insights for motor insulation system design.

## 1. Introduction

With the rapid development of the automobile industry, the market share of new energy vehicles has increased markedly [1]. As the core component of new energy vehicles, the performance of the motor directly determines the overall vehicle performance [2,3]. Due to their high slot fullness and power density, hair-pin motors are widely used in the manufacturing of new energy vehicles. In the manufacturing process of hair-pin motor stators, stator drip coating is a key step; its quality directly affects the insulation performance of the stator. Common motor insulation materials are mostly polymeric resin lacquers, such as polyester lacquers, epoxy lacquers, and polyurethane lacquers [4,5]. These resins are thermosetting polymers that, upon curing, form cross-linked structures which are infusible and insoluble [6]. When selecting insulating varnish materials, both the insulating properties and the environmental impact, specifically the emission of volatile organic compounds (VOCs), must be considered [7,8]. VOCs in polyester resins are typically released during the polymerization or curing process by toxic crosslinking monomers. These monomers act as solvents for the base polyester to reduce its viscosity and are reactive during the curing process [9]. This issue can be avoided by using solvent-free insulating varnishes. Another critical factor in selecting insulation materials is thermal performance. According to international standards [7], thermal classification testing specifies the maximum temperature that the motor can withstand during operation. Unsaturated polyester imide resins, due to the introduction of imide functional groups, offer higher thermal ratings and improved insulating properties [10]. Among these, unsaturated polyester imide resin (UPIR) is often used as the main material for motor stator insulation because of its superior insulating properties, mechanical strength, and thermal stability. However, during the curing process of resin composite insulating varnish, due to the dissimilar material properties between the insulating varnish and the stator copper conductor, residual stresses are generated at the interface [11,12,13,14], resulting in cracks or gaps in the insulating varnish and partial discharges, which can seriously affect the motor’s lifespan.

Numerous studies have focused on analyzing the curing residual stresses in resin composites, particularly regarding their composition and other characteristics. However, relatively few studies have examined the interfacial residual stresses during the curing process of stator insulating varnish in hair-pin motors. Dewangan et al. employed X-ray diffraction (XRD) techniques to study the curing of polyester resins under high pressure [15]. The results were validated through three-dimensional numerical simulations, and a method to increase porosity was proposed. Hao et al. used fiber Bragg grating (FBG) technology to detect cure residual strains in the E51/W93 epoxy resin system with varying Al_2_O_3_ content. Their primary focus was on examining the effect of Al_2_O_3_ content on gel temperature and gel time, and they proposed a method to simultaneously detect temperature and curing residual strain using thermocouple temperature compensation combined with an FBG sensor [16]. Yang et al. designed a device to measure the elastic modulus of epoxy resin potting materials based on a viscoelastic model; they derived an expression for the viscoelastic intrinsic properties of epoxy resins [17]. Wang et al. identified the cracking issues in insulating surfaces caused by curing stresses during the secondary curing of epoxy resins, and investigated the impact of curing agent content on curing stresses [18]. Qu et al. investigated the curing stresses of modified epoxy resins with varying toughener contents. They analyzed the material properties of the epoxy resin curing process and curing residual stresses using differential scanning calorimetry (DSC), Dynamic Mechanical Analysis (DMA), Fourier-Transform Infrared Spectroscopy (FTIR), and Thermogravimetric Analysis (TGA). The curing thermal stresses were calculated using the Stoney equation [19].

The above studies primarily focus on the analysis of the residual stresses during the curing process of resin systems, with a particular emphasis on the effects of the material composition and curing parameters. These studies utilize techniques such as DSC and FBG to measure the curing-induced residual strain and stress under various conditions, highlighting the importance of precise stress measurement and control. By investigating the curing characteristics of resin materials, they offer valuable insights into the stress mitigation and crack prevention in insulating materials. This body of work underscores the critical role of the material properties and curing conditions in influencing the residual stresses and mechanical integrity, and it has inspired the dual-FBG curing strain experiment in this study. However, these studies predominantly focus on single-resin systems or simple geometries, leaving a gap in understanding the residual stresses at complex interfaces, such as those in motor stators. This gap highlights the necessity of the present research, which aimed to investigate the interfacial residual stresses during the curing process of insulating varnish in hair-pin motor stators.

Based on previous studies, the numerical simulation method can be regarded as a universal approach for stress analysis during the curing process of resin materials. Ye et al. conducted a multi-physics numerical simulation of L-shaped composite laminates, developing a model that incorporates the resin’s transient flow compaction to predict the curing behavior of resin composites [20]. Balaji et al. combined the data from DSC experiments with neural networks to predict the deformation of resin materials during curing. They verified their model’s accuracy through finite element simulations, which demonstrated greater realism than the conventional curing kinetics models at temperature rise rates below 3 K/min [21]. Wu et al. studied the deformation of polyurethane composites during the curing when used as battery bezels, focusing on the factors affecting curing deformation; withholding pressure was identified as the most significant factor [22].

The above studies demonstrate the value of numerical simulation in analyzing the curing behavior of resin systems. They show that numerical methods are highly effective for predicting the residual stresses and deformation during curing, improving the accuracy and efficiency of stress analysis. Additionally, combining simulations with experimental validation has proven to be a reliable way to understand the curing process under different conditions and have provided important guidance for this research. Building on these insights, this study applies numerical simulation to investigate the curing behavior of insulating varnish in hair-pin motor stators, with a focus on addressing the residual stress issues at complex geometric interfaces.

The simulation and analysis of the curing residual stresses in resin-insulating varnish, one of the most commonly used motor-insulating materials, are crucial. Numerical simulation not only reduces the experimental costs but also predicts the curing defects caused by various factors and assesses their impact on the electrical performance of motors. In this paper, we focus on the residual stresses, temperature, and strain during the curing process of stator-insulating varnish for hair-pin motors and explore the characteristics and mechanisms of the curing residual stresses through a combination of simulation and experimental verification.

**Figure 1:**
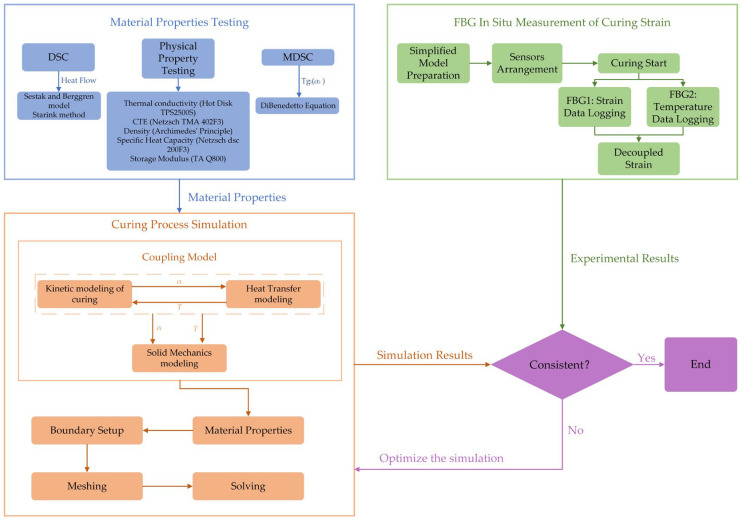
Flowchart of the experimental and simulation process.

This paper will follow the process outlined in Figure 1.

This study presents a novel approach to analyze the curing residual stresses at the conductor–insulator interface of hairpin motors. Research on the curing residual stresses of unsaturated polyester imide resin insulating varnishes is limited. Additionally, unlike the traditional methods using thermocouple temperature compensation, this study uniquely employs encapsulated FBG for temperature monitoring, which leads to more accurate experimental results. Furthermore, by considering the temperature or curing degree dependence of the material properties, a three-dimensional simulation model is developed. The model established in this study can be applied to the residual stress analysis during the curing process of other thermoset resin materials, thus contributing to a better understanding of the residual stress generation behavior of resin-based insulating materials in various fields.

## 2. FBG In Situ Measurement of Curing Strain

### 2.1. Materials

The insulating varnish used in this study is a one-component, low-volatility impregnating varnish, Voltatex^®^ 4200 (Axalta Coating Systems Ltd., Philadelphia, PA, USA). The main component is an unsaturated polyester imide resin, which offers excellent insulating properties for the motor stator. According to the official performance specifications, Voltatex^®^ 4200 demonstrates a dielectric strength of 85 kV/mm at 155 °C, significantly higher than that of comparable epoxy resin insulating varnishes, which typically range from 20 to 30 kV/mm [23,24]. Furthermore, the thermal class of unsaturated polyester imide resin materials is generally higher than that of epoxy resin-based insulating varnishes [4]. Additionally, UPIR has a lower initial viscosity compared to typical epoxy resins, which is more favorable for the contact between the insulation varnish and the insulated surface during the motor insulation process.

### 2.2. Principle of the FBG Sensor

The FBG sensor is a single-mode fiber that detects reflections at specific wavelengths as light passes through the FBG fiber [25]. The Bragg wavelength expression is presented in Equation (1):

(1) $$\lambda_{B}=2n_{eff}\Lambda$$

where $\lambda_{B}$ is the wavelength reflected by the Bragg grating, $n_{eff}$ is the effective reflection coefficient of the fiber, and Λ is the grating period.

FBG is sensitive to external changes, and for FBG sensors affected by both temperature and strain, $\lambda_{B}$ is linearly correlated with changes in temperature and strain. The Bragg wavelength shift can be expressed as:

(2) $${\Delta \lambda }_{B}=k_{\varepsilon }\varepsilon +k_{T}\Delta T$$

where $k_{\varepsilon }$ is the coefficient related to strain and the photo-elastic coefficient, and $k_{T}$ is the coefficient related to the thermal-optic coefficient and thermal expansion coefficient of the optical fiber.

Due to the characteristics of unsaturated polyester imide resin, in the early stage of insulating varnish curing, the varnish exists as a viscous liquid. Upon reaching the gel point, the molecules gradually crosslink, and the resin hardens into a solid. At this stage, the shrinkage strain caused by curing and the thermal expansion and contraction due to temperature changes can be transferred to the FBG sensor. The curing strain of the resin can then be determined by monitoring the change in the wavelength of the FBG.

### 2.3. Experimental Design

**Figure 2:**
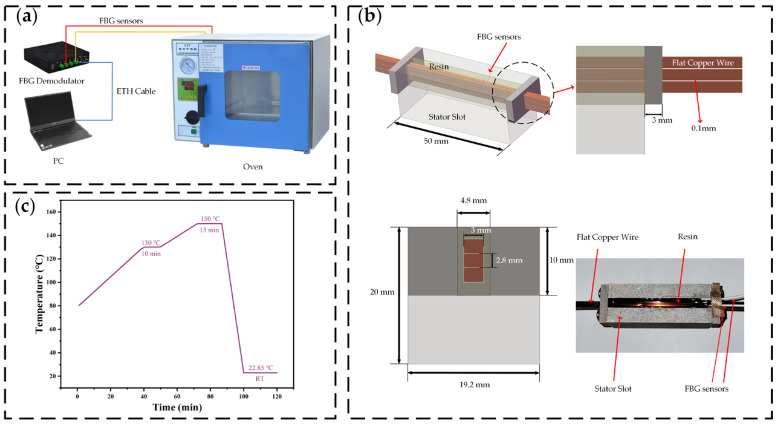
FBG in situ measurement. (**a**) Test equipment; (**b**) curing test model; (**c**) curing procedure.

Equation (2) clearly shows that when using FBG to measure the strain influenced by both temperature and strain, temperature compensation must be applied. In this experiment, the temperature compensation is provided by a packaged FBG temperature sensor, which offers more accurate real-time temperature readings. The wavelength changes due to strain are obtained through the fiber Bragg grating demodulator. The test equipment is illustrated in Figure 2a.

The FBG sensor used is the OSC-1100 (Xi’an Huance Automation Co., Ltd., Xi’an, China). The FBG demodulator is the HOSI-1000E (Huaying Instrument Equipment Co., Ltd., Xi’an, China). The oven model is DZF-6020 (Shanghai Yiheng Scientific Instrument Co., Ltd., Shanghai, China).

The high slot fullness of the hair-pin motor’s stator is attributed to the geometry of the flat copper wire and the winding method, which allows the stator slots to be filled. The stator conductor is made of copper, while the stator core (including the stator slots) is composed of silicon steel, which offers superior magnetic properties and lower energy loss. The motor consists of 8 strands of conductors, which can provide higher power.

In this paper, to simplify the model, only the stresses generated at the interface of the flat copper wires by the insulating varnish between the three conductors are investigated. The simplified model is illustrated in Figure 2b.

The slot structure surrounding the copper wire is made of silicon steel and simulates the stator slots. During the experiment, insulating varnish is filled into the groove. After ensuring complete immersion, two FBG sensors are inserted on either side of the copper wire to measure temperature and strain data during the curing process of the insulating varnish at the same location.

Figure 2c illustrates the curing process of the drop-dip test. First, the stator must be heated to 80 °C, after which the insulating varnish is drop-dipped onto it. The temperature is then increased from 80 °C to 130 °C and held for 10 min, during which the curing reaction takes place. Subsequently, the temperature is raised to 150 °C and held for 15 min to cure the insulating varnish fully and eliminate thermal stress. Finally, the stator is cooled to room temperature to complete the curing process.

It should be noted that some sources of error may arise during the experiment. First, since a packaged FBG is used for temperature compensation, it is essential to ensure that the insulating varnish does not seep into the encapsulation tube. Second, environmental vibrations or fiber bending may cause signal fluctuations or distortion [26]. Third, misalignment of the optical fiber could result in minor measurement deviations. These factors were carefully considered during the experimental setup, and measures such as sensor calibration and environmental stabilization were implemented to minimize their impact.

### 2.4. Experimental Results and Analysis

As described in Section 2.2, the FBG sensor is affected by both temperature and strain. When using the FBG sensor to measure the temperature, it is essential to measure without applying force. Teflon capillary-encapsulated FBGs are used to compensate for the temperature effects on strain. Two FBGs are placed symmetrically on the left and right sides of the flat copper wire for the test. From Equation (2), by knowing the sensitivity coefficients $k_{\varepsilon }$ and $k_{T}$, and the temperature change ΔT of the optical fiber, the true cure strain ε can be determined according to Equation (3):

(3) $$\varepsilon =\frac{{\Delta \lambda }_{B}-k_{T}\Delta T}{k_{\varepsilon }}$$

where the sensitivity coefficients $k_{\varepsilon }$ and $k_{T}$ of the optical fiber are provided by the manufacturer, and the temperature change ΔT is obtained from the FBG sensor used for temperature compensation.

**Figure 3:**
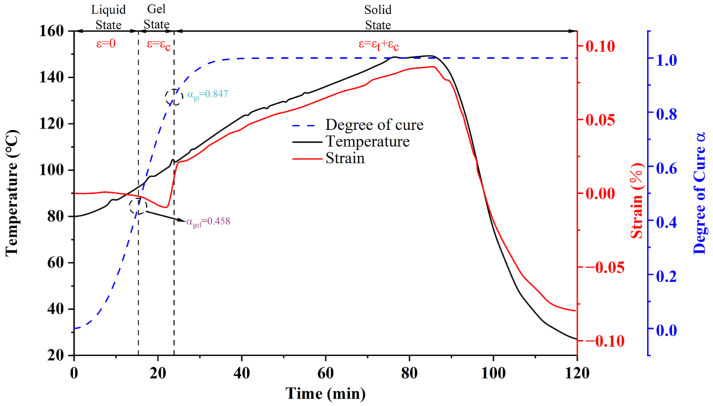
Curing strain test results of insulating varnish.

The stress and temperature test results for the insulating varnish curing process are illustrated in Figure 3. The initial strain value was set in the FBG demodulator’s PC software Hippo 3.1.4 when the specimen reached 80 °C, and the curing degree curve was later obtained from the curing kinetics model analysis. The figure shows that there is no significant change in the strain during the early stage of curing, as the resin remains in a flow state at this point. Due to preheating to 80 °C, the resin’s viscosity decreases significantly, but cross-linking has not yet occurred. The molecular chains mainly rely on physical interactions, allowing the internal molecules to move freely.

When the strain curve decreases significantly, it indicates that the resin has undergone considerable volume contraction during curing. Since the primary component of the material is unsaturated polyester imide resin, the curing process is accompanied by significant contraction. Even though both thermal strain and chemical contraction occur simultaneously, chemical contraction predominates, resulting in the observed decrease in the strain curve. When the strain curve begins to rise, it indicates that the material has gradually transitioned from a viscous-fluid state to a glassy state. Most cross-linking reactions within the material have been completed, and the mobility of the molecular chains is significantly reduced. Due to the further temperature increase, the material undergoes thermal expansion, causing the strain curve to rise.

During the heat preservation stage and the subsequent warming stage, the temperature profile does not fully align with the preset temperature profile. One reason for this is that the resin’s curing reaction is exothermic, and the heat released during curing leads to a local temperature increase. Another reason is that the stator slot has a low thermal conductivity, causing slow heat transfer from the outside, which results in noticeable thermal hysteresis.

In the post-temperature rise stage, the strain and temperature curves are roughly linear because the curing reaction of the insulating varnish is nearly complete, and the strain is primarily affected by thermal strain. However, the strain and temperature curves are not completely linear because the coefficient of thermal expansion (CTE) of the cured resin exhibits a noticeable nonlinear characteristic with changing temperature. The coefficient of thermal expansion of the cured resin was measured to be 45.93 × 10^−^⁶/K at 25 °C, increasing to 152.55 × 10^−^⁶/K at 150 °C, as measured by the Netzsch TMA 402F3 (Netzsch-Gerätebau GmbH, Selb, Germany). Another reason is that the higher crosslink density of the material hinders molecular chain movement, slowing the release of internal stresses [27,28].

During cooling, when the strain curve of the resin decreases more slowly than the temperature curve, it indicates that the material is passing through the glass transition temperature (${\;T}_{g}$). At this point, the material transforms from a rubbery state to a glassy state, further restricting molecular chain movement and hampering the release of internal stresses. Thus, a partially nonlinear relationship between the strain curve and the temperature curve is observed during the cooling phase. As discussed in Section 3.1, the glass transition temperature of this insulating varnish after complete curing is 97 °C, which aligns with the figure.

## 3. Three-Dimensional Numerical Simulation of the Curing Process

Due to the compact structure of the stator flat-wire winding, measuring the data related to the curing process of the insulating varnish is challenging, especially as the distance between the flat wires is only 0.1 mm. This small gap limits the ability of sensors to detect the curing residual stress directly. Numerical simulation provides an effective solution to this problem. The simulations in this study were performed using COMSOL Multiphysics. The simulation of the insulating varnish curing process involves multi-physical field coupling, including the temperature field, curing degree field, and stress–strain field. The relevant mathematical model includes a heat conduction model, a curing kinetics model, and a mechanical model of the material.

**Figure 4:**
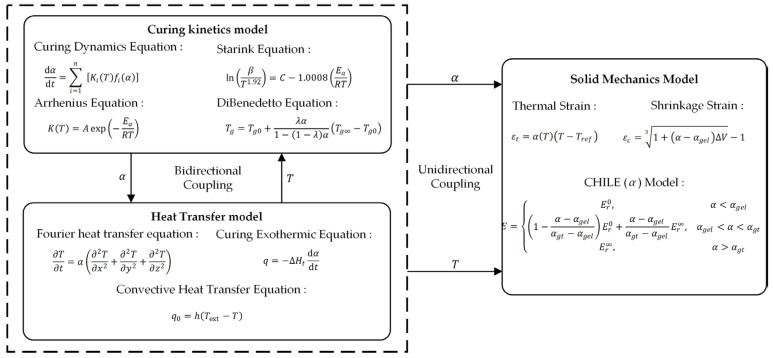
Coupling relationship.

The temperature field and the curing field are bi-directionally coupled. By solving the relationship between the degree of curing and temperature, the calculated temperature and degree of curing are coupled with the stress–strain field to predict the curing deformation of the insulating varnish and the interfacial residual stresses. The coupling relationship is illustrated in Figure 4.

It should be noted that the distribution of insulating varnish is assumed to be uniform during the curing of drip varnish on flat-wire windings. Since the insulating varnish coating is thin and the curing time is short, the resin exhibits almost no mobility during curing. Therefore, the effect of the flow compaction model can be neglected.

### 3.1. Curing Kinetics Model

#### 3.1.1. Data Analysis and Model Derivation

The cure rate of insulating varnish reflects the dynamic behavior of the curing process. There are two approaches to studying this process: a macroscale phenomenological model and a microscale mechanistic model [29]. The phenomenological model considers only the overall process of the reaction, and a single rate equation, *dα*/*dt*, is used to express this model. In contrast, the mechanistic model can better predict the curing process and explain the reaction mechanism. However, due to the complexity of the insulating varnish curing reaction, the mechanistic model requires more parameters to accurately describe the curing process, leading to a significant increase in the computational demands and making it difficult to obtain a highly accurate model. Therefore, in this paper, we adopt the phenomenological model, expressed as Equation (4) [30,31,32,33]:

(4) $$\frac{d\alpha }{dt}=\sum_{i=1}^{n}\left[K_{i}\left(T\right)f_{i}\left(\alpha \right)\right]$$

where α is the degree of cure, dα/dt is the curing rate, *T* is the temperature, and $K_{i}(T)$ and $f_{i}\left(\alpha \right)$ represent the reaction rate equation and the curing reaction equation, respectively. The reaction rate equation $K\left(T\right)$ is parameterized by the Arrhenius formula shown in Equation (5) [34].

(5) $$K\left(T\right)=A\;exp\left(-\frac{E_{a}}{RT}\right)$$

where T, A, $E_{a},$ and R represent the temperature, pre-exponential factor, activation energy, and the molar gas constant, respectively.

The primary component of the insulating varnish is unsaturated polyester imide resin, and the degree of curing is commonly used to describe the extent of the resin’s curing reaction. The degree of curing is a key parameter in simulating the curing process, as it significantly affects the final performance of the resin material (e.g., mechanical strength, chemical resistance, and thermal stability). To develop a kinetic model for the resin’s curing process, the curing behavior was analyzed using differential scanning calorimetry (DSC) in this study.

**Figure 5:**
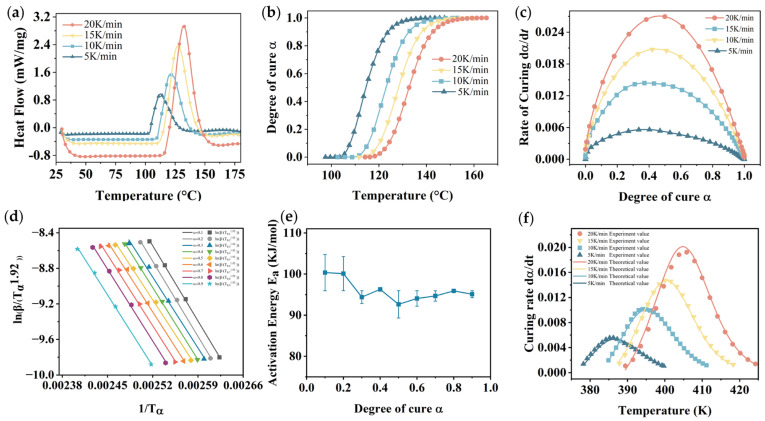
DSC data analysis. (**a**) Heat flow–temperature curves; (**b**) degree of cure–temperature curves; (**c**) rate of curing at different degrees of cure; (**d**) fitting curves of the Starink equation; (**e**) reaction activation energy $E_{a}$ at different degrees of cure; (**f**) model validation curves.

Four samples were heated from 25 °C to 180 °C at heating rates of 5 K/min, 10 K/min, 15 K/min, and 20 K/min using a Netzsch DSC 200F3. The sample mass ranged from 7 to 10 mg, and the nitrogen flow rate was set at 50 mL/min. The heat flow-temperature curves obtained by DSC are illustrated in Figure 5a. (Note: the heat flow curve obtained by the device is exothermic downward; it was multiplied by −1 to display the exothermic peak upward).

The curing onset temperature $T_{i}$, peak temperature $T_{p}$, termination temperature $T_{f}$, and total heat of reaction $\Delta H_{t}$ obtained for the curing process of the insulating varnish samples at different heating rates are presented in Table 1.

It is observed that the exothermic peak of the curing reaction shifts significantly to the right as the heating rate of the insulating varnish increases. The curing onset temperature $T_{i}$, peak temperature $T_{p}$, and termination temperature $T_{f}$ also increase significantly with the higher heating rate. Equation (6) can be used to calculate the exothermic quantity $\Delta H\left(t\right)$ of the curing reaction. This integral represents the area under the heat flow–time curve and the baseline from the onset to the end of the curing reaction.

(6) $$\Delta H\left(t\right)=\int_{0}^{t}\phi \left(t\right)\cdot dt$$

where $\phi \left(t\right)\;$ is the heat flow function.

The total exothermic heat of curing for the insulating varnish remained relatively consistent across different heating rates, with an average total heat of reaction $\Delta H_{t}$ = 192.625 $J\cdot g^{-1}$.

The complete curing of the insulating varnish is achieved when the reaction exotherm $\Delta H\left(t\right)$ equals the total reaction heat $\Delta H_{t}$. Therefore, the degree of cure α at any given time can be defined as the ratio between the exothermic heat $\Delta H\left(t\right)$ and the total reaction heat $\Delta H_{t}$ from the beginning of the curing reaction to a specific moment, as shown in Equation (7).

(7) $$\alpha \left(t\right)=\frac{\Delta H\left(t\right)}{\Delta H_{t}}$$

Figure 5b presents the relationship between the degree of cure and temperature. It exhibits a distinct S-shaped curve, where the cure rate increases initially and then decreases, consistent with the autocatalytic model [35,36,37].

To further validate the results, the time derivative of the degree of cure was calculated to obtain the curing rate. Figure 5c illustrates the relationship between the curing rate dα/dt and the degree of cure $\alpha \left(t\right)$.

It is evident that, at various heating rates, the curing rate first increases and subsequently decreases as the degree of cure increases. The peak of the curing rate occurs at approximately a degree of cure of 0.4, further indicating that the curing of this insulating varnish conforms to the self-catalytic model [38].

The curing process of insulating varnishes is usually influenced by two factors: curing kinetics control and diffusion control. According to the studies in the literature [39], at the beginning of the isothermal cure, the curing rate of the resin is controlled solely by the curing kinetics, and with an increasing degree of cure, the material becomes partially glassy, hindering the cross-linking reaction within the resin; thus, the process becomes diffusion-controlled. According to previous thermal analysis methods [39,40], the first step involves determining the activation energy of the cure reaction and assessing whether a single model can describe the curing kinetics by analyzing the relationship between the activation energy and the degree of cure. Commonly used methods for calculating the activation energy include the Starink model [41,42], the Kissinger model [42], the Ozawa model [43,44], with the Starink model considered more accurate compared to other model-free isotropic methods [45].

By fitting the experimental data with the Starink equation shown in Equation (8), the activation energy ($E_{a}$) of the insulating varnish reaction can be determined.

(8) $$\begin{array}{c} \ln\left(\frac{\beta }{T^{1.92}}\right)=C-1.0008\left(\frac{E_{a}}{RT}\right) \end{array}$$

where β represents the rate of ramping, T denotes the reaction temperature, C is a constant, $E_{a}$ stands for the reaction activation energy, and R refers to the molar gas constant.

According to the Starink equation, $\ln\left(\beta /T^{1.92}\right)$ was linearly fitted against 1/T for different heating rates, with the results presented in Figure 5d.

The obtained slopes of the curves for various degrees of cure and the corresponding reaction activation energies are shown in Table 2. The R-squared values for different degrees of cure are all greater than 0.99, indicating a strong fit. The average reaction activation energy ($E_{a}$) is calculated to be 95.96 kJ/mol.

The curing process of insulating varnish resin is controlled by two mechanisms: reaction control and diffusion control. According to the study in [39], when the activation energy required for the reaction changes significantly with the degree of cure, it indicates that the reaction is controlled by diffusion. In this case, using a single model to describe the reaction would lead to errors, and the reaction can only be described using a model that includes a diffusion term [46,47,48] (such as the Kamal model [49]). According to previous thermal analysis methods [40], the activation energy of the curing reaction must first be determined, and the relationship between the activation energy and degree of cure should be examined to determine whether a single model can describe the curing kinetics.

The reaction activation energies at different curing degrees are presented in Figure 5e, and the activation energy ($E_{a}$) remains relatively constant once the degree of cure exceeds 0.5. Therefore, it can be concluded that the curing behavior of this insulating varnish is controlled solely by the reaction kinetics.

There are multiple models for describing the curing kinetics of autocatalytic composites. Notably, the Prout–Tompkins (PT) model [50,51,52,53] incorporates diffusion terms, thereby providing a more accurate depiction of the cure front propagation under conditions of high fiber content or complex thermal conduction. This makes the PT model particularly effective in capturing the autocatalytic characteristics of the reaction. However, the PT model is associated with high computational complexity. In contrast, the Sestak and Berggren (SB) model [54] offers a simplified description of the reaction mechanism, resulting in enhanced computational efficiency. Consequently, the SB model is well suited for large-scale or complex structural numerical simulations within specific resin systems and processing conditions. Therefore, in this paper, we chose to use the SB model to determine the curing kinetics of insulating varnishes. By combining the temperature-rate-dependent equation of Sestak and Berggren with the classical autocatalytic reaction model, the fitted curing kinetics model, represented by Equation (9), was obtained.

(9) $$\begin{array}{c} \frac{d\alpha }{dt}=Aexp\left(-\frac{E_{a}}{RT}\right)\alpha^{m}{\left(1-\alpha \right)}^{n} \end{array}$$

where A represents the pre-exponential factor, and m,  n are the reaction orders.

The average reaction activation energy ($E_{a}$) obtained by the Starink method was substituted into Equation (5) and fitted using the least squares method to derive the kinetic parameters presented in Table 3.

Ultimately, the kinetic model equation for the curing of this insulating paint can be expressed as Equation (10):

(10) $$\begin{array}{c} \frac{d\alpha }{dt}=e^{25.69085}exp\left(-\frac{95955.45}{RT}\right)\alpha^{0.44}{\left(1-\alpha \right)}^{1.25} \end{array}$$

To verify the model’s accuracy, the curing rate–temperature curves obtained from the model at different heating rates were compared with the experimentally obtained curves, as shown in Figure 5f. The model curves showed a higher degree of consistency with the experimental curves.

The glass transition temperature ($T_{g}$) is one of the most important thermodynamic parameters in the study of thermosetting resins [31]. When a material exceeds its glass transition temperature, its physical and chemical properties change significantly. Previous studies have demonstrated that the glass transition temperature increases as the degree of cure increases. Therefore, the glass transition temperatures of insulating varnishes at various degrees of cure were tested using modulated DSC, and DiBenedetto’s equation was applied to identify these temperatures.

(11) $$\begin{array}{c} T_{g}=T_{g0}+\frac{\lambda \alpha }{1-\left(1-\lambda \right)\alpha }\left(T_{g\infty }-T_{g0}\right)\; \end{array}$$

where $T_{g0}$ represents the glass transition temperature of the uncured state, $T_{g\infty }$ denotes the glass transition temperature of the fully cured state, and λ is the fitting parameter.

**Figure 6:**
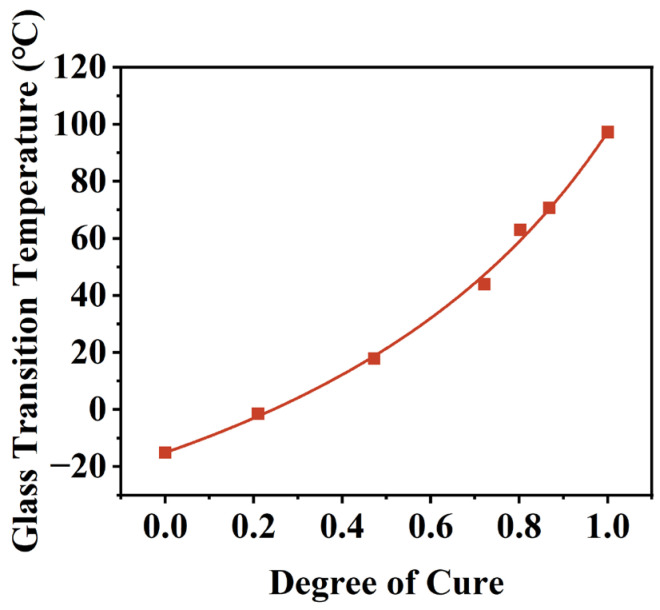
Fitting curve of the DiBenedetto equation.

First, five samples were thermostated at 90 °C for different durations and then rapidly cooled to −40 °C. The degree of cure was determined from the exothermic peak of the irreversible heat flow curve using MDSC. The samples were then heated from −40 °C to 200 °C at a rate of 3 K/min with a temperature amplitude of 1 K and a period of 60 s. The glass transition temperature ($T_{g}$) of each sample was determined from the post-elevated reversible heat flow curve, following ASTM E1356 (3 K/min, inflection point method). The relationship between the glass transition temperature and curing of insulating varnish determined by Equation (11) is shown in Figure 6.

The equation fitting yields an $R^{2}$ value of 0.99735, indicating a high degree of fit. The fitted parameter λ is 0.48, suggesting that the molecular chain segments of the insulating varnish possess a certain degree of mobility, resulting in a relatively low sensitivity of the glass transition temperature to the degree of cure.

#### 3.1.2. Boundary Setup

In the simulation software, the ODE and Differential-Algebraic Equation (DAE) modules, in conjunction with the Heat Transfer module, can be used to solve the temperature-dependent curing kinetics equations by inputting the expression of Equation (1) directly into the ODE module.

The default equation form of the ODE module is Equation (12):

(12) $$\begin{array}{c} e_{a}\frac{\partial^{2}alpha}{\partial t^{2}}+d_{a}\frac{\partial alpha}{\partial t}=f \end{array}$$

where $e_{a}$ is the mass coefficient, $d_{a}$ is the damping coefficient, and f is the source term equation. In comparison to Equation (1), $e_{a}$ is set to 0 and $d_{a}$ is set to 1, and f is set to $e^{25.69085}exp\left(-\frac{95955.45}{RT}\right)\alpha^{0.44}{\left(1-\alpha \right)}^{1.25}$.

The initial value of the degree of cure is set to 1 × 10^−20^, which helps prevent errors in the solver caused by computing the negative exponent of zero.

### 3.2. Heat Transfer Model

During the insulation curing process of the hair-pin motor stator, the heating device provides the thermal energy required for curing the insulating resin. Owing to the differing thermal conductivities of the flat copper wire and insulating varnish, the varnish is not heated uniformly. Since the curing process is exothermic, it further exacerbates the temperature non-uniformity. In addition, the material properties of the insulating varnish evolve with the degree of cure, and these factors collectively contribute to the generation of residual stresses and strains during the curing process.

#### 3.2.1. Model Derivation and Parameter Determination

The thermal field during the insulating varnish curing process is essentially a nonlinear heat transfer problem with an internal heat source. This problem arises from the exothermic reaction of the unsaturated polyester imide resin curing in the insulating varnish [55]. This nonlinear heat transfer problem can be characterized by the Fourier heat transfer equation coupled with the curing exothermic Equation (13).

(13) $$\begin{array}{c} \begin{array}{c} \rho C_{p}\frac{dT}{dt}=\frac{d}{dx}\left(K_{xx}\frac{dT}{dx}\right)+\frac{d}{dy}\left(K_{yy}\frac{dT}{dy}\right) \end{array}+\frac{d}{dz}\left(K_{zz}\frac{dT}{dz}\right)+\rho \Delta H_{t}\frac{d\alpha }{dt} \end{array}$$

where ρ represents the density, $C_{p}$ is the specific heat, T is the temperature, $\Delta H_{t}$ is the total reaction heat, α is the degree of cure, and $K_{xx},\;\;K_{yy}$ and $K_{zz}$ represent the thermal conductivities in the three directions.

Assuming that the insulating varnish material is isotropic, the thermal conductivities in all three directions are equal. This equation can be solved through the coupling of the Heat Transfer module with the Curing Kinetics module.

Thermal conductivity, specific heat capacity, and density are important material properties of insulating varnish materials. The properties of insulating varnish obtained from experimental measurements are presented in Table 4.

The material properties of the insulating varnish are linearly related to the degree of cure, and the relationship between these parameters and the degree of cure can be expressed by Equation (14) [56].

(14) $$\left\{\begin{array}{l} k(\alpha )=k_{a}(1-\alpha )+k_{b}\alpha \\ C_{P}(\alpha )=C_{Pa}(1-\alpha )+C_{Pb}\\ \rho (\alpha )=\rho_{a}(1-\alpha )+\rho_{b}\alpha \end{array}\alpha \right.$$

where subscript a represents the parameters before curing, and subscript b represents the parameters after curing.

#### 3.2.2. Boundary Setup

During the curing process, an external oven heats the insulating varnish, and the heat transfer between the insulating varnish and the hot air occurs through natural convection, with the convective heat transfer described by Equation (15).

(15) $$q_{0}=h\left(T_{ext}-T\right)$$

where $q_{0}\;$ is the heat flux, h is the convective heat transfer coefficient, $T_{ext}$ is the external temperature, and T is the temperature of the insulating varnish; $T_{ext}$ can be defined as the heating temperature via a custom function.

During the drop-dip curing process, the insulating varnish directly comes into contact with the flat copper wires and heats them together, so the heat flux boundaries are all the boundaries in contact with the outside during the numerical simulation. The convective heat transfer coefficient h is set to 25 $W/(m^{2}\cdot K)$ based on an assumption. The heat source is insulating paint, and the heat source expression is the rightmost term $\rho \Delta H_{t}d\alpha /dt$ in Equation (11). And the reference temperature is set to a preset value of 80 °C.

### 3.3. Solid Mechanics Model

During the curing process, the material properties of the insulating varnish change with the degree of cure and temperature, leading to the generation of internal stresses at the interface between the flat copper wire and the insulating varnish. The curing process of the insulating varnish material undergoes a viscous flow state, a highly elastic state, and a glassy state. Cure shrinkage occurs during the highly elastic stage. During the cooling stage, the varnish shrinks unevenly due to structural differences, cooling rates, and the mismatch in the coefficients of thermal expansion of varnish and the stator, resulting in the formation of internal stresses, thereby affecting the insulation performance.

#### 3.3.1. Model Derivation and Parameter Determination

The strain during the curing process of insulating varnish is composed of two parts: one part is the thermal strain induced by heat, and the other part is the shrinkage strain caused by the curing of the resin material, as represented in Equation (16):

(16) $$\varepsilon =\varepsilon_{t}+\varepsilon_{c}$$

where ε represents the total strain, $\varepsilon_{t}$ denotes the thermal strain, and $\varepsilon_{c}$ refers to the cure shrinkage strain.

Studies have shown that the shrinkage of resin materials during curing occurs during the glassy state [18,29].

The thermal strain $\varepsilon_{t}$ is a function of the temperature and the coefficient of thermal expansion, as expressed in Equation (17):

(17) $$\varepsilon_{t}=\alpha \left(T\right)\left(T-T_{ref}\right)$$

where $\alpha \left(T\right)$ represents the coefficient of thermal expansion of the material at temperature T and $T_{ref}$ denotes the reference temperature.

**Figure 7:**
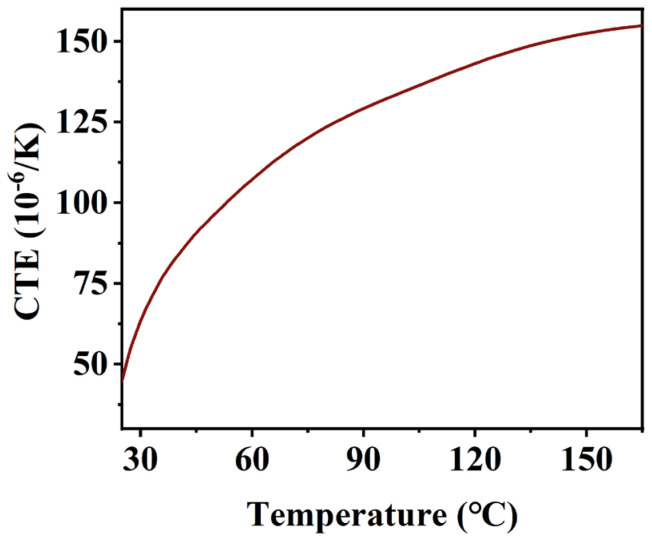
Thermal expansion coefficient curve of cured insulating varnish.

The coefficient of thermal expansion of the insulating varnish after curing can be measured using a linear thermal expansion meter, with the results presented in Figure 7.

The coefficient of thermal expansion throughout the curing process is given by Equation (18) [57]:

(18) $$\alpha_{CTE}\left(T,\alpha \right)=\left\{\begin{array}{l} \alpha_{CTE}^{v},\;\;\;\;\alpha <\alpha_{gel}\\ \alpha_{CTE}^{r},\;\;\;\;\alpha \geq \alpha_{gel},\;\;\;\;T\geq T_{g}(\alpha )\\ \alpha_{CTE}^{g},\;\;\;\;\alpha \geq \alpha_{T_{g}},\;\;\;\;T<T_{g}(\alpha ) \end{array}\right.$$

where $\alpha_{CTE}^{v},\;\;\alpha_{CTE}^{r},$ and $\alpha_{CTE}^{g}$ represent the thermal expansion coefficients for the viscous state, rubbery state, and glassy state, respectively, $\alpha_{gel}$ denotes the degree of cure at the gel point, and $\alpha_{T_{g}}$ represents the degree of cure at the glass transition point.

Assuming that the insulating varnish is isotropic, the shrinkage is uniform in all directions during the curing process. The following equation can describe the chemical shrinkage strain of the resin:

(19) $$\varepsilon_{c}=\sqrt[{3}]{1+\Delta v}-1$$

where $\varepsilon_{c}$ represents the curing strain, and Δv denotes the volume change rate.

As shrinkage strain occurs mainly after the gel state; it can be expressed as a function of the degree of cure and total volume change ΔV, as shown in Equation (20):

(20) $$\Delta v=\left(\alpha -\alpha_{gel}\right)\Delta V$$

where α represents the current degree of cure and $\alpha_{gel}$ denotes the degree of cure at the gel point.

Thus, the expression for the chemical shrinkage strain of the insulating varnish can be derived, as shown in Equation (21) [58].

(21) $$\varepsilon_{c}=\sqrt[{3}]{1+\left(\alpha -\alpha_{gel}\right)\Delta V}-1$$

The linear elastic constitutive model is commonly used to predict the curing deformation of composite materials. Its general form is given by:

(22) $$\left\{\sigma \right\}=\left[E\right]\left(\left\{\varepsilon \right\}-\left\{\varepsilon_{0}\right\}\right)+\left\{\sigma_{0}\right\}$$

where $\left\{\sigma \right\}$ and $\left\{\sigma_{0}\right\}$ represent the stress and initial stress to be solved, respectively; $\left[E\right]$ is the stiffness matrix; $\left\{\varepsilon \right\}$ and $\left\{\varepsilon_{0}\right\}$ are the total strain and initial strain in tensor form.

During the curing process of insulating resin materials, the material properties evolve, depending on both temperature and curing degree. Consequently, the formula in Equation (22) cannot fully capture the material behavior during curing. To address this limitation, the CHILE (Cure Hardening Instantaneous Linear Elastic) model is introduced, which incorporates the time-dependent characteristics of the elastic modulus to account for the dynamic changes in the material properties throughout the curing process.

Bogetti et al. proposed the CHILE (α) model, based on the curing degree, which assumes that the modulus of the resin varies linearly with the curing degree [58]. This model describes the evolution of the elastic modulus during the curing of composite materials by considering the material properties at different curing stages. The fundamental stress–strain relationship in this model is given by Equation (23):

(23) $$\left\{\sigma \right\}=\left[Q\left(\alpha ,T\right)\right]\left\{\varepsilon \right\}$$

where $\left[Q\left(\alpha ,T\right)\right]$ is the time-dependent stiffness matrix, which is a function of both temperature and curing degree.

The Poisson’s ratio ν of the insulating varnish is an important parameter in the stiffness matrix. To this end, uniaxial tensile tests were conducted on fully cured insulating varnish samples according to the ASTM D638 standard, and the deformation was measured using strain gauges. The measured Poisson’s ratio of the fully cured insulating varnish was 0.37. It has been found that the variation in Poisson’s ratio during curing has a negligible impact on the residual stress accumulation [59,60,61]. Therefore, in this study, for the sake of model simplification, the Poisson’s ratio of the insulating varnish is assumed to be 0.37.

The change in the modulus during the curing process of insulating varnish can be divided into three stages: In the first stage, the epoxy resin behaves as a fluid with a low storage modulus. In the second stage, as the curing reaction progresses, the insulating varnish transitions from a viscoelastic state to a gel state, and then to a glassy state. The storage modulus in this stage is a function of the curing degree. In the third stage, after the curing reaction is complete, when the temperature is below the glass transition temperature, the resin undergoes minimal deformation under external forces. The storage modulus becomes large and remains almost constant. In this study, we used the CHILE (α) model to describe the modulus of elasticity of the resin during curing, E, as shown in Equation (24) [58,62]:

(24) $$E=\left\{\begin{array}{cc} E_{r}^{0}, & \alpha <\alpha_{gel}\\ \left(1-\frac{\alpha -\alpha_{gel}}{\alpha_{gt}-\alpha_{gel}}\right)E_{r}^{0}+\frac{\alpha -\alpha_{gel}}{\alpha_{gt}-\alpha_{gel}}E_{r}^{\infty }, & \alpha_{gel}<\alpha <\alpha_{gt}\\ E_{r}^{\infty }, & \alpha >\alpha_{gt} \end{array}\right.$$

$E_{r}^{0}$ and $E_{r}^{\infty }$ represent the modulus of elasticity of the resin before and after curing, $\alpha_{gel}$ and $\alpha_{gt}$ denote the gel point cure and glass point cure of the resin.

**Figure 8:**
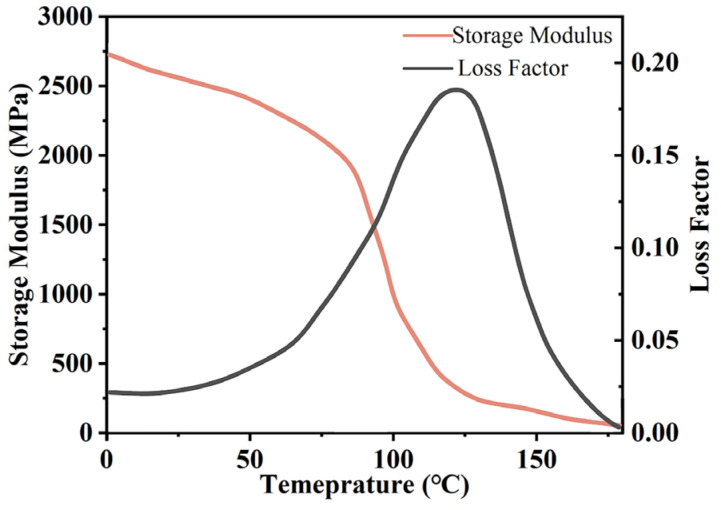
DMA test results.

Since the viscosity of the insulating varnish is not considered, the storage modulus is used as a substitute for the elastic modulus and it was determined through DMA using a TA Q800 instrument. The test was conducted at a frequency of 1 Hz with a three-point bending setup, and with a heating rate of 3 °C/min from 0 °C to 200 °C, and the resulting storage modulus and loss factor are shown in Figure 8. The insulating varnish was determined to have $E_{r}^{0}$ = 2.7 MPa and $E_{r}^{\infty }$ = 2729 MPa.

#### 3.3.2. Boundary Setup

Considering only the insulating varnish curing process on a single copper wire during the stator dripping process, the surface between the copper wire and the insulating varnish is set as a continuous boundary, with a fixed constraint applied on the other side of the copper wire. In the simulation software, the solid heat transfer and solid mechanics nodes can be used to simulate the thermal expansion of the cured insulating varnish. However, since the total strain ε during curing is the sum of the thermal strain $\varepsilon_{t}$ due to thermal expansion and the contraction strain $\varepsilon_{c}$ due to the curing of the insulating varnish, it is necessary to add an external strain sub-node under the solid mechanics–linear elastic material node. Equation (21) is incorporated into this node to simulate the cure shrinkage strain. In addition, to improve the convergence of the model, the “free rigid body inhibition” node can be added. This node functions to prevent the solid mechanics module from generating multiple non-converging solutions, and the deformation results from the simulation with this node better align with the real deformation results.

### 3.4. Simulation Setup

**Figure 9:**
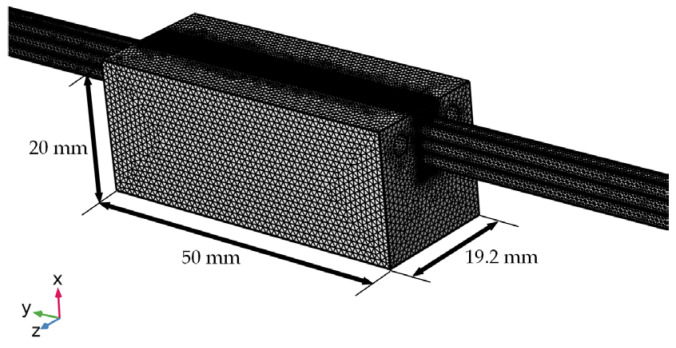
Finite element mesh for the simplified curing module.

The simulation was conducted using COMSOL’s automatic tetrahedral meshing. Differentiated mesh sizes were applied to the copper conductor, insulating varnish, and external stator slot regions. To ensure higher resolution at the interface between the insulating varnish and copper conductor, this region was assigned a maximum element size of 0.1 mm and a minimum of 0.02 mm, while the external stator slot used a maximum size of 0.4 mm and a minimum of 0.2 mm. The final mesh contained 2,112,621 elements. The mesh result is shown in Figure 9.

The glass transition temperature of the material was determined using the DiBenedetto equation. The parameters for the equation, including λ, were fitted using glass transition temperatures obtained at various curing degrees through MDSC experiments. In the simulation, the glass transition temperature before curing ($T_{g0}$), after full curing ($T_{gu}$), and the parameter λ were defined.

To model the material behavior during curing, the elastic modulus and the coefficient of thermal expansion (CTE) were defined based on the curing state of the insulating paint. The CTE after curing was modeled as a temperature-dependent function, fitted to the experimental thermal expansion data using an analytical function in the software. Additionally, the heat capacity, thermal conductivity, and density were defined as functions of the degree of cure, consistent with the assumptions outlined in the manuscript.

For the curing shrinkage, the curing strain was defined as a function of the degree of cure based on Equation (21). This strain was implemented as an external strain in the solid mechanics module to simulate the effects of curing shrinkage. The curing volumetric shrinkage was calculated based on the density change before and after curing.

The simulation in this study uses a step-by-step solution approach. The transient solver in COMSOL is applied to solve the bidirectional coupling of the curing kinetics model and the heat transfer model. Then, the steady-state solver is used to inherit the solution from the transient solver to calculate the solid mechanics model.

Given the spatial effects involved in the curing process, a 3D model was employed to ensure accurate simulation. A 2D model would not capture the asymmetry of the stator slots or the heat transfer characteristics effectively. The use of a 3D model provides a more realistic representation of the curing process. While the results are presented in 2D plots, they clearly show the temperature and stress distribution within the stator slots.

## 4. Results Discussion

Using the aforementioned physical field setup and the material properties obtained from experimental data, a Multiphysics-coupled simulation under the same conditions as the experiment is conducted. The temperature and strain results from both the experiment and simulation are compared to analyze the stress–strain behavior during the insulating varnish curing process.

### 4.1. Simulation Results Validation

**Figure 10:**
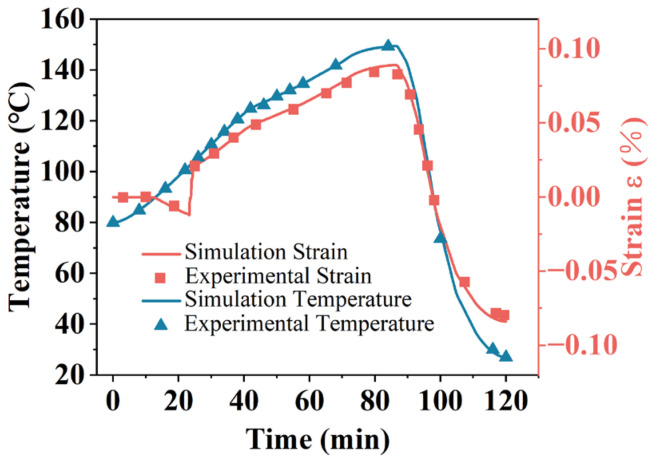
Comparison of strain and temperature simulation curves with experimental data.

The temperature–time curve and strain–time curve of the simulation results were compared with the experimental values, as shown in Figure 10. The comparison indicates that the simulation model can generally reflect the strain and temperature conditions of the actual curing process of the insulating paint. Some data points do not fully match the experimental data because the strain test curve was obtained through decoupled calculations, and approximations in the calculation process may have affected some values. Additionally, some material properties were derived from empirical formulas, which may not correspond to the actual values during the curing process. These discrepancies can be addressed through subsequent optimization of the model.

### 4.2. Results Analysis

**Figure 11:**
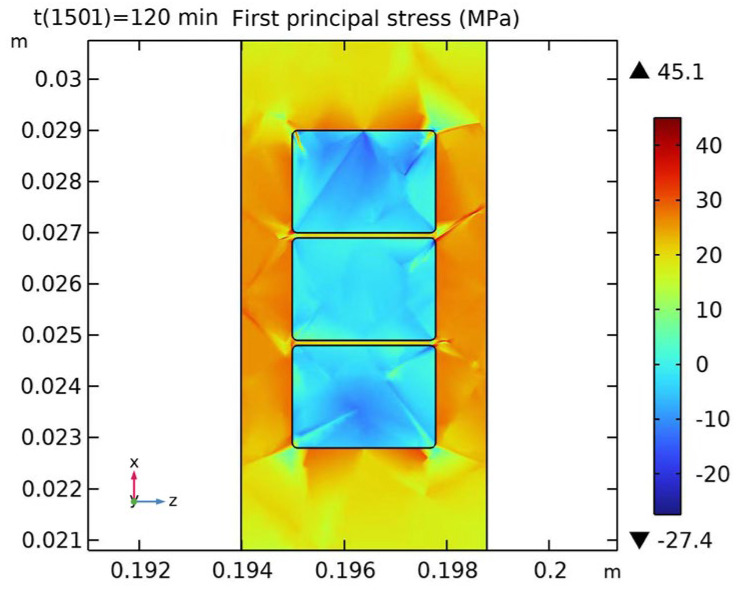
Stress distribution in the cross-section of copper wire and insulating varnish.

Figure 11 shows the stress distribution of the copper wire cross-section at the end of curing and after cooling to room temperature. The figure illustrates that the cured insulating varnish experiences the highest stress at the interface with the copper wire, reaching 45.1 MPa, with a difference of nearly 40 MPa compared to the minimum stress within the insulating varnish, indicating a stress concentration phenomenon. This is a key factor contributing to insulation defects.

**Figure 12:**
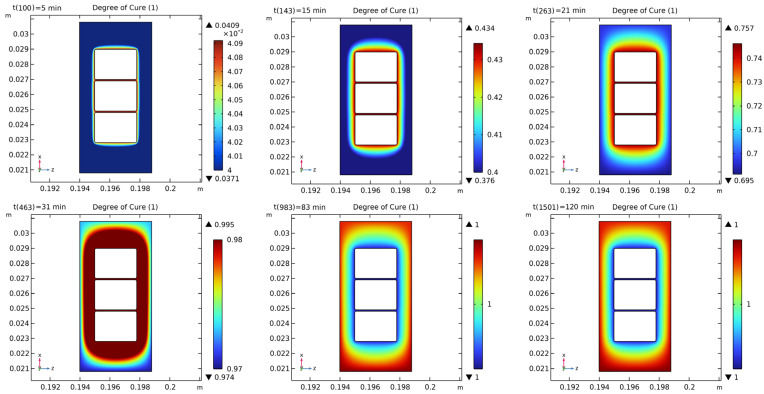
Degree of cure distribution in the cross-section of copper wire and insulating varnish.

**Figure 13:**
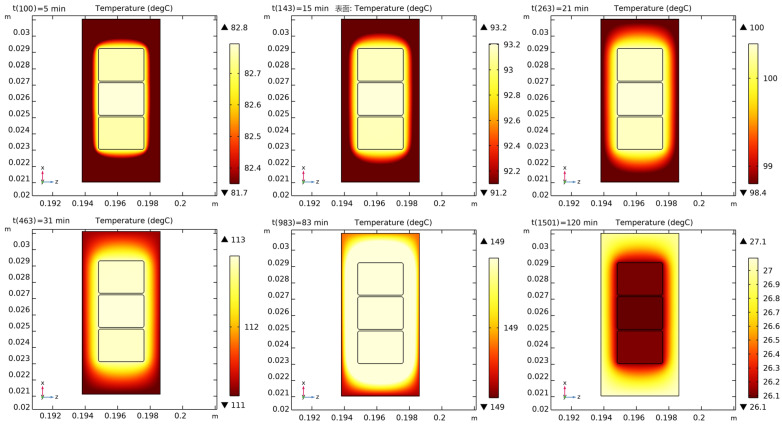
Temperature distribution in the cross-section of copper wire and insulating varnish.

Figure 12 and Figure 13 show the distribution of the curing degree and temperature in the initial stage, mid-stage, complete curing, and final stage of the insulating varnish curing process.

In the initial stage of curing, the temperature of the insulating varnish near the copper wires rises first due to the high thermal conductivity of the copper, allowing the varnish to reach the external preset temperature more quickly. During the 15–31 min, the varnish begins curing, with a temperature distribution that is lower at the edges and higher in the center. By 83 min, when the external preset temperature reaches 150 °C and has been maintained for almost 10 min, the temperature distribution becomes more uniform. At this point, the varnish is nearly fully cured, and the temperature is close to the preset value. In the cooling phase after 83 min, the varnish near the copper wire cools faster, similar to the heating phase.

The curing and crosslinking of the varnish are temperature driven, so the distribution of the curing degree is similar to that of the temperature. As the temperature increases, the varnish near the copper wire begins to cure first, with the curing degree following a stepwise pattern. By 31 min, the curing reaction weakens or disappears, and the curing degree is nearly complete. Although the temperature starts to drop after 83 min, the low thermal conductivity of the varnish causes a noticeable thermal lag during both the heating and cooling phases. As a result, the temperature and curing degree distributions align more closely, reaching a steady state and indicating that the curing process is nearing completion.

The strains in the curing process of insulating varnish primarily consist of the shrinkage strain generated during curing and the thermal strain caused by temperature changes. While the material composition influences the shrinkage strain, the thermal strain is affected by the material’s thermal expansion coefficient and the temperature difference. This paper presents a preliminary study of the effect of the cure temperature on the cure stress.

**Figure 14:**
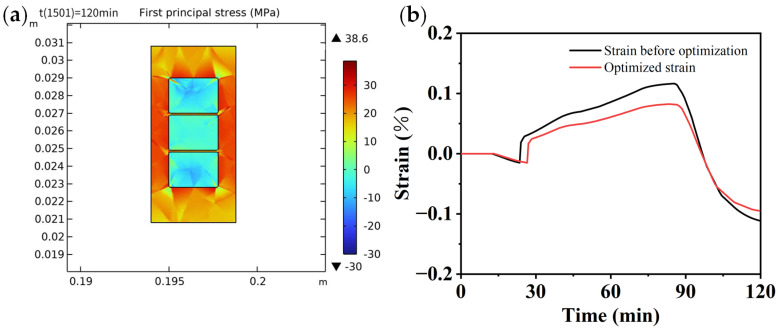
(**a**) Stress distribution; (**b**) strain curve of measurement points.

The curing process was modified by adjusting the maximum temperature of the post-rise phase to 130 °C. The resulting stress distribution is shown in Figure 14a. The results show that when the maximum cure temperature is reduced to 130 °C, the maximum stress value decreases to 38.6 MPa, representing a 14.4% reduction.

Figure 14b shows the strain curves of the measured points after optimization. Following the reduction in the post-heating stage temperature, the glass transition of the insulating varnish exhibits hysteresis, and the strains observed during and at the end of curing are smaller than those before optimization. This indicates that the post-heating stage temperature has a significant effect on the residual stress.

## 5. Conclusions

In this study, the curing process of insulating varnish for a hair-pin motor stator was monitored with respect to temperature and strain using FBG sensors. The curing kinetics of the insulating varnish material was investigated, and a simulation model of the process was developed using the finite element method, leading to the following conclusions:

(1) Both strain and temperature influence FBG sensors, and the temperature can be monitored using packaged FBG sensors. By analyzing the wavelength shift due to temperature changes, it is possible to decouple the strain variations. Through non-isothermal differential scanning calorimetry (DSC) experiments, the curing kinetics model of Voltatex^®^ 4200 insulating varnish, primarily composed of unsaturated polyimide resin, was investigated. The curing reaction of this insulating varnish is characterized as a self-catalyzed reaction with a relatively large pre-exponential factor, allowing for rapid curing. Additionally, a comparison was conducted between the curing kinetics model obtained using the Starink method and the experimental data, which demonstrated a high degree of consistency.

(2) Based on the material properties of the insulating varnish after curing and the DSC test data, a three-dimensional simulation model of the curing process was established to analyze the temperature, degree of cure, and stress distribution. Given that the thermal conductivity of the flat copper wire is significantly higher than that of the resin and the silicon steel stator slot, there is a pronounced hysteresis phenomenon in heat transfer within the stator slot during curing. The temperature and degree of cure of the insulating varnish near the flat copper wire are greater than those near the stator slot.

In terms of the strain field, thermal strain is the primary influencing factor as curing nears completion, while shrinkage strain predominates during the initial stages of curing. The temperature and degree of cure of the insulating varnish near the flat copper wire are higher than those near the stator slot. Regarding the strain field, the strain in the insulating varnish during the initial curing phase is primarily shrinkage strain. In contrast, thermal strain becomes the primary influencing factor as curing approaches completion.

(3) After curing, the maximum stress occurs at the interface between the flat copper wire and the insulating varnish, with a maximum stress value of 45.1 MPa. There is a significant phenomenon of stress concentration, which is a major factor contributing to insulation defects. By modifying the temperature during the post-heating stage and using the established simulation model for stress prediction, the results indicate that the temperature of the post-heating stage also affects the residual stress. Specifically, when the maximum temperature during the post-heating stage is reduced to 130 °C, the maximum residual stress decreases by 14.4%.

However, there are some limitations to the present study that should be considered for future research:

(1) The relationships between density, constant pressure specific heat, and thermal conductivity with the degree of cure are based on assumptions, which may limit the accuracy of the predictions.

(2) The influence of flow consolidation, which could affect the material’s behavior during curing, was not incorporated into the simulation model.

(3) The simulation model employs a modified linear elastic constitutive model to represent the material’s behavior, which replaces the viscoelastic model. This simplification might not fully capture the time-dependent behavior of the material during the curing process.

(4) The long-term effects, such as thermal fatigue, were not specifically investigated. Thermal fatigue can be a critical factor in materials subjected to cyclic thermal loading, as the mismatched thermal expansion between the copper wire and the insulating varnish can lead to further stress accumulation and degradation over time. This could affect the material’s long-term performance and reliability, which warrants further investigation in future research.

## Figures and Tables

**Table 1:** Characteristic temperature and total reaction heat at different heating rates.

Heating Rate (K/min)	$T_{i}$ (°C)	$T_{p}$ (°C)	$T_{f}$ (°C)	$\Delta H_{t}(J\cdot g^{-1})$
5	103.6	112.8	127.8	198.2
10	110.2	121.0	135.8	187.2
15	114.5	127.4	141.3	192.5
20	118.4	132.1	145.8	192.6

**Table 2:** Data fitted by the Starink method.

Degree of Cure α	Slope	$E_{a}\;(kJ/mol)$	R-Squared
0.1	−12,080.9	100.3607	0.99956
0.2	−12,050.2	100.1052	0.99912
0.3	−11,364.3	94.40691	0.99532
0.4	−11,590.8	96.28893	0.99952
0.5	−11,152.3	92.64607	0.99761
0.6	−11,324	94.07278	0.99747
0.7	−11,398.3	94.68944	0.99751
0.8	−11,545.9	95.91561	0.99851
0.9	−11,449.3	95.11346	0.99884

**Table 3:** Kinetic parameters at different heating rates.

Heating Rate β (K/min)	lnA	m	n	R-Squared
5	25.6134	0.35678	1.22978	0.99772
10	25.72109	0.44207	1.26424	0.99917
15	25.72341	0.47535	1.24681	0.99984
20	25.70551	0.50076	1.26355	0.99992
Average	25.69085	0.44374	1.251095	0.99916

**Table 4:** Thermodynamic parameters of insulating varnish.

Material Properties	Unit	Before Curing	Cured
Thermal Conductivity $k\left(\alpha \right)$	W/(m·K)	0.21	0.23
Specific Heat Capacity $C_{p}(\alpha )$	J/(kg·K)	1908	1273
Density ρ(α)	$kg/m^{3}$	1.12	1.169

## Data Availability

The original contributions presented in this study are included in the article. Further inquiries can be directed to the corresponding authors.
